# A Cross-Sectional Study of the Distribution Patterns and Potential Determinants in Plasma Selenium Status Among Chinese Adults With Hypertension

**DOI:** 10.3389/fnut.2022.882309

**Published:** 2022-05-17

**Authors:** Zhuo Wang, Tengfei Lin, Yaping Wei, Yun Song, Lishun Liu, Ziyi Zhou, Xiao Huang, Ping Chen, Chengzhang Liu, Youbao Li, Binyan Wang, Jianping Li, Yan Zhang, Yong Huo, Hao Zhang, Xiping Xu, Xianhui Qin, Huiyuan Guo

**Affiliations:** ^1^Key Laboratory of Precision Nutrition and Food Quality, Ministry of Education, Department of Nutrition and Health, China Agricultural University, Beijing, China; ^2^Shenzhen Institutes of Advanced Technology, Chinese Academy of Sciences, Shenzhen, China; ^3^Institute of Biomedicine, Anhui Medical University, Hefei, China; ^4^Department of Cardiology, The Second Affiliated Hospital of Nanchang University, Nanchang, China; ^5^College of Pharmacy, Jinan University, Guangzhou, China; ^6^National Clinical Research Center for Kidney Disease, State Key Laboratory for Organ Failure Research, Division of Nephrology, Nanfang Hospital, Southern Medical University, Guangzhou, China; ^7^Shenzhen Evergreen Medical Institute, Shenzhen, China; ^8^Department of Cardiology, Peking University First Hospital, Beijing, China

**Keywords:** selenium, distribution patterns, hypertension, geographical, demographics, lifestyle, nutritional epidemiology

## Abstract

Selenium (Se) is an essential trace element in selenoproteins biosynthesis for the human body and plays an important role in the prevention and control of subsequent cardiovascular disease in adults with hypertension. However, reports on Se status and its potential determinants in populations from different regions of China are limited, especially data on adults with hypertension, a high-risk group more vulnerable to oxidative stress. Thus, we conducted a cross-sectional study from February 2017 to May 2018 of 2,599 participants (1,389 men and 1,210 women) on middle-aged to elderly adults with hypertension with a mean age of 63.1 years (SD 13.3) from 14 provinces of China and aimed to examine the relationship of plasma Se status with demographic characteristics and lifestyles. Overall, the male participants (mean value 75.0 μg/L) tended to have higher plasma Se concentrations than the female participants (73.7 μg/L) when controlling for relevant factors. There were significant differences among regions, and in age and body mass index (BMI) in plasma Se distribution, and plasma Se concentrations were significantly lower among those in the regions with relatively lower Se, aged 60 years or older, and with BMI lower than 28 kg/m^2^. Moreover, a higher frequency of meat consumption (1–2 or ≥3 times/week vs. <1 time/week) was significantly associated with higher plasma Se concentrations in men and women, and male alcohol drinkers had significantly higher plasma Se concentrations than non-alcohol drinkers. Adequate consumption of fruits and vegetables (0.5–1.5 kg/week) was associated with higher plasma Se concentrations among women, but was associated with relatively lower plasma Se concentrations in men. Our results indicated relatively low plasma Se status in Chinese adults with hypertension from 14 provinces, while specific factors including geographic, demographic, and lifestyle characteristics and blood pressure were significantly associated with plasma Se status in this hypertensive population. In addition, more studies are required to further evaluate dietary structure and other lifestyle factors that influence circulating Se status.

## Introduction

Selenium (Se) is an essential trace element for human health and plays an important role in the body *via* selenoproteins (1). Se is an integral part of the activation site in the form of selenocysteine (Sec) for glutathione peroxidase (GPX) (2), an enzyme that protects tissues against oxidative stress and catalyzes the reduction of peroxidase following cellular damage. Se can be metabolized to Sec and incorporated into selenoprotein P (SELENOP) (3), which is secreted into the plasma. SELENOP is involved in male fertility, proper brain functioning, and other biological processes. A U-shaped relationship between Se status and chronic disease has been previously reported (4). The different amounts of environmental exposure may have a significant impact on human Se status, especially because dietary Se bioavailability is mostly governed by environmental factors such as Se concentration in the soil, water, and air (5).

A few studies have reported that blood pressure is associated with multiple metal biomarkers (6). However, the relationship between Se and hypertension and subsequent chronic diseases is still inconclusive (7). Our previous findings from the China Stroke Primary Prevention Trial (CSPPT) indicated that there is a lower risk of first stroke in adults with hypertension who have relatively higher plasma Se concentrations, which suggests the importance of maintaining proper nutritional status to prevent cardiovascular diseases (CVDs) (8). Adults with hypertension may be more vulnerable to the effects of oxidative stress, and more sensitive to environmental exposure levels of Se and the antioxidant effects of Se-dependent GPX (9). The prevalence of hypertension in China is high and rising, and a variety of CVDs induced by hypertension have substantial socioeconomic consequences (10). Thus, comprehensively revealing the characteristics and determinants of Se distribution in a population with hypertension, especially in those exposed to various environmental Se levels, will contribute to developing nutrition intervention strategies in populations with hypertension for lowering the disease burden.

China is one of the 40 countries designated by the World Health Organization (WHO) as having low or insufficient levels of Se. Low soil Se levels are widespread, and low dietary sources of Se and unhealthy dietary habits may raise the potential risk of Se deficiency in the Chinese population. It is especially important that attention be paid to the Se status of Chinese people, given that the soil Se deficiency in China is growing because of climate change (11). People with high blood pressure tend to have lower Se levels than healthy subjects, according to previous studies based on limited sample sizes in China and the former Yugoslavia (12, 13). However, this relationship could differ by location as seen in results from the United States and Denmark where hypertensive individuals were found to have higher Se levels (14, 15). Due to the numerous determinants of Se levels, the Se distribution among adults with hypertension remains unconfirmed. The association of the environment, demographics, and dietary habits with Se status among Chinese adults with hypertension especially needs more evidence. Furthermore, most prior investigations used more conveniently obtained samples to assess Se status, such as nails, hair, or urine (16–18). However, the above indicators do not reflect circulating Se because plasma and serum Se are considered the most suitable indicators in epidemiological studies (19). Although the previous studies have shown adverse effects of Se exposure at low levels, the debate concerning the issue of selenoprotein optimization is still ongoing for the evidence regarding the need to increase Se levels is still controversial (20, 21). In particular, high Se exposure has been shown to be related to an increased risk of type 2 diabetes (22). Thus, more evidence is needed to reveal the optimal Se status for humans. Overall, most of the previous research has indicated a relatively low Se status in Chinese with Keshan disease or Kashin–Beck disease (23). However, there is still a lack of large-scale and representative reports on Se status in Chinese adults with hypertension.

In general, previous studies have not adequately disclosed the relationship between the environment, demographic characteristics, and lifestyle with Se status in Chinese adults with hypertension. Thus, we conducted a cross-sectional survey of randomly selected adults with hypertension in 14 Chinese provinces, using inductively coupled plasma mass spectrometry (ICP–MS) detection methods to characterize plasma Se concentration. By evaluating the distribution of plasma Se status and its potential determinants of Se among adults with hypertension, we aimed to identify populations at high risk for low Se and the potential strategies for the adjustment of Se status.

## Materials and Methods

### Study Population

We conducted a multi-centric, non-interventional, observational, real-world study for the identification and registration of a high-risk population with both hypertension and elevated total homocysteine levels (tHcy ≥ 10 μmol/L) within China, which was initiated in February 2017. Patients were recruited from the community through open recruitment rather than through random selection. The inclusion criteria included the following individuals: (1) Individuals who had hypertension, defined as seated, resting systolic blood pressure (SBP) of 140 mm Hg or higher, or diastolic blood pressure (DBP) of 90 mm Hg or higher at the recruitment visit, or who were taking an antihypertensive medication, according to the diagnostic criteria of the 2,010 Chinese guidelines for the management of hypertension (24); (2) Individuals who had elevated total homocysteine (tHcy ≥ 10 μmol/L); and (3) individuals who gave signed, written, and informed consent.

Of the eligible participants in this observational study, two subsamples were selected, without duplication, at 10 and 16 months after the study began, in December 2017 and June 2018, respectively. First, 900 participants were randomly selected from nine provinces and stratified by province, who were enrolled from June to August 2017 and had complete screening records (physical exam, questionnaire, and biological samples). Second, another 1,709 participants were randomly selected from 14 provinces (including the 9 provinces in the first sampling set plus an additional 5 provinces) and stratified by province, sex, and age groups, who were enrolled from February 2017 to May 2018 and had complete screening records (25). Finally, after combining the two subsamples, a total of 2,599 participants from all 14 provinces without duplication, were included in our current study, excluding those with missing values of Se (Supplementary Figure 1).

The study protocol was approved by the Ethics Committee of Peking University First Hospital, Beijing, China (Approval No.: 20161231), and the study was conducted according to the Declaration of Helsinki. All methods were performed in accordance with the relevant guidelines and regulations. Written, informed consent was obtained from all the participants.

### Blood Sample Collection and Laboratory Assays

A fasting, venous blood sample was obtained from each participant. Plasma samples were separated within 30 min of collection and stored at −80°C. Plasma Se concentrations of the randomly selected participants were measured by ICP–MS using Thermo Fisher iCAP Q ICPMS, in a commercial lab (Beijing DIAN Medical Diagnostics Laboratory, Beijing, China). Both intra-assay and inter-assay coefficients of variation (CV) for duplicate samples (randomly placed among the study sample) were calculated. The intra-assay CV of plasma Se ranged from 2.14 to 9.38%, while the inter-assay ranged from 2.41 to 3.31%.

### Major Definitions

The 14 provinces were stratified by soil Se concentrations based on reports from previous studies, and these were then categorized into three levels: Se-marginal (0.125– <0.175 mg/kg), Se-sufficient (0.175 to <0.40 mg/kg). and Se-rich (0.40–3.0 mg/kg) (26, 27). The Se-marginal regions in China for this study included four study provinces (Gansu, Heilongjiang, Liaoning, and Shandong), while Se-sufficient regions included eight study provinces (Anhui, Beijing, Hebei, Jiangsu, Ningxia, Yunnan, Shanxi, and Sichuan), and Se-rich regions included two study provinces (Guangxi and Hunan).

### Statistical Analysis

For baseline characteristics and plasma Se concentrations, normally distributed and approximately normally distributed continuous variables were presented as mean ± SD and were compared using *t*-tests, and non-normally distributed continuous variables were presented as median (25th–75th percentile) and were compared using the Wilcoxon–Rank–Sum tests (for two factors) or the Kruskal–Wallis tests (for more than two factors). Categorical variables were presented as numbers (percentage) and were compared using the χ^2^ (for two factors) tests or the ANOVA tests (for more than two factors). Pairwise comparisons of average plasma Se concentrations in each group were conducted by least significant difference (LSD) tests.

Adjusted Se values were calculated by general linear regression models after controlling for sex, age, body mass index (BMI), smoking status (never, past, current: defined as having smoked ≥1 cigarette per day or ≥18 packs in the past year), alcohol drinking status (never, past, current: defined as drinking alcohol at least 2 times per week in the past year), region (Se-marginal, Se-sufficient, and Se-rich provinces in China), SBP, DBP, history of hypertension (no, yes), antihypertensive drug use (no, yes), multivitamin use (no, yes), meat consumption (<1 time/week, 1–3 times/week, 3–5 or more times/week), and consumption of fruits and vegetables (<0.5 kg/week, 0.5–1.5 kg/week, >1.5 kg/week). In addition, the linear trends in Se concentration across age, BMI, region, smoking, alcohol drinking, meat consumption, and fruit and vegetable consumption groups were assessed using general linear regression models.

Regression models were estimated *via* ordinary least squares and restricted cubic-spline functions were used to assess non-linear trends with three knots for continuous variables stratified by sex. Beta coefficients (βs) and 95% confidence intervals (CIs) of Se concentrations in association with selected demographic variables were estimated using linear regression models, without or with adjustment for potential confounding factors.

The map of provincial distribution patterns of the prevalence of plasma Se was plotted using R software, version 3.6.1.^1^ The SHP file of the China map was obtained from the website of the Resource and Environment Data Cloud Platform.^2^

A two-tailed *p* < 0.05 was considered statistically significant in all analyses. R soft-ware, version 3.6.1 (see text footnote 1) and Empower version 2.0^3^ were used for all statistical analyses and map plotting.

## Results

### Study Participants and Baseline Characteristics

As illustrated in the flowchart of the study participants (Supplementary Figure 1), the current study included a total of 2,599 participants, among which 736 (28.3%) were from Se-marginal regions of China, 1,463 (56.3%) were from Se-sufficient regions of China, and 400 (15.4%) were from Se-rich regions of China (Table 1).

**TABLE 1:** Baseline characteristics of the study participants stratified by region.

Characteristics	Se-marginal region^1^	Se-sufficient region	Se-rich region
*N*	736	1,463	400
Male, *n* (%)	383 (52.0)	779 (53.2)	227 (56.8)
**Age, years**			
<60	306 (41.6)	587 (40.1)	163 (40.8)
60 to <70	178 (24.2)	373 (25.5)	113 (28.2)
≥70	252 (34.2)	503 (34.4)	124 (31.0)
**Body mass index, kg/m^2^**			
<24	242 (32.9)	550 (37.6)*	202 (50.5) ^††^
24 to <28	317 (43.1)	648 (44.3)	147 (36.8)
≥28	177 (24.0)	265 (18.1)	51 (12.8)
**Smoking status, *n* (%)**			
Never	528 (71.7)	1,006 (68.8)**	286 (71.5)
Past	42 (5.7)	202 (13.8)	27 (6.8)
Current	166 (22.6)	255 (17.4)	87 (21.8)
**Alcohol drinking status, *n* (%)**			
Never	564 (76.6)	1072 (73.3)*	312 (78.0) ^†^
Past	32 (4.3)	126 (8.6)	33 (8.2)
Current	140 (19.0)	265 (18.1)	55 (13.8)
SBP, mm Hg	147.0 (135.0–156.1)	139.3 (130.0–150.0) **	143.2 (133.0–153.3) ^†^
DBP, mm Hg	90.0 (80.7–97.0)	85.7 (78.7–92.3) **	86.8 (81.3–92.3) ^†^
Antihypertensive drug use, *n* (%)	557 (75.7)	1,102 (75.3)	99 (24.8) ^††^
Multivitamin use, *n* (%)	52 (7.1)	148 (10.1) *	9 (2.2) ^††^
History of hypertension, *n* (%)	592 (80.4)	1423 (97.3) **	352 (88.0) ^†^
**Meat consumption frequency, *n* (%)**			
<1 time/week	183 (24.9)	326 (22.3)**	84 (21.0) ^††^
1–2 times/week	273 (37.1)	596 (40.7)	94 (23.5)
3–5 or more times/week	280 (38.0)	541 (37.0)	222 (55.5)
**Fruits and vegetables consumption, *n* (%)**			
<0.5 kg/week	183 (24.9)	326 (22.3)	84 (21.0) ^††^
0.5–1.5 kg/week	273 (37.1)	596 (40.7)	94 (23.5)
>1.5 kg/week	280 (38.0)	541 (37.0)	222 (55.5)
Se, μg/L	69.3 ± 20.7	74.0 ± 19.3 **	85.1 ± 18.5 ^††^

*For continuous variables, the values are presented as median interquartile range (IQR) or mean ± SD.*

*^1^Se-marginal areas in China include four provinces (Gansu, Heilongjiang, Liaoning, and Shandong), Se-sufficient areas in China include eight provinces (Anhui, Beijing, Hebei, Jiangsu, Ningxia, Shanxi, Sichuan, and Yunnan), and Se-rich areas in China include two provinces (Guangxi and Hunan).*

*^2^Plasma Se concentrations were adjusted for sex, age, BMI, SBP, DBP, history of hypertension, antihypertensive drug use, multivitamin use, smoking, alcohol drinking, meat consumption, and consumption of fruits and vegetables.*

*p < 0.05, **p < 0.001; the values significantly differed among the participants from Se-marginal and Se-sufficient areas in China.

*^†^p < 0.05, ^††^p < 0.001; the values significantly differed among the participants from Se-marginal and Se-rich areas in China.*

Overall, this study included relatively more men (53.4%), elderly participants aged 60 years or above (59.4%), and those with normal BMI (42.8%) among all the participants (Supplementary Table 1). Comparisons of the baseline characteristics of the participants randomly selected in the current analysis and those not included, showed no obvious differences, and are presented in Supplementary Table 2. The participant characteristics by region are listed in Table 1. The participants in Se-sufficient regions and Se-rich regions had lower baseline blood pressure (systolic and diastolic) and fewer were current smokers and alcohol drinkers, who had lower BMI and a higher history of hypertension than those from Se-marginal regions. The participants from Se-rich regions had higher frequencies of meat consumption and fruit and vegetable consumption than those from Se-marginal regions and Se-sufficient regions. Moreover, multivitamin use was low across all regions. Overall, compared with the participants from Se-marginal regions, significantly higher average plasma Se concentrations were found in the participants from Se-sufficient regions and Se-rich regions (*p* < 0.05) after adjustment for the related factors. Moreover, the participant characteristics stratified by the 14 study provinces and sex are listed in Supplementary Tables 1, 3. The mean ± SD values of adjusted plasma Se concentrations were higher in the male participants (75.0 ± 19.4, μg/L) than in the female participants (73.7 ± 19.4, μg/L) (Supplementary Table 3). Accordingly, as shown in Supplementary Figure 2, compared with women, there was a shift toward the right in the distribution of plasma Se concentration in males.

### Demographic and Lifestyle Distribution Patterns of Plasma Selenium Status

**FIGURE 1:**
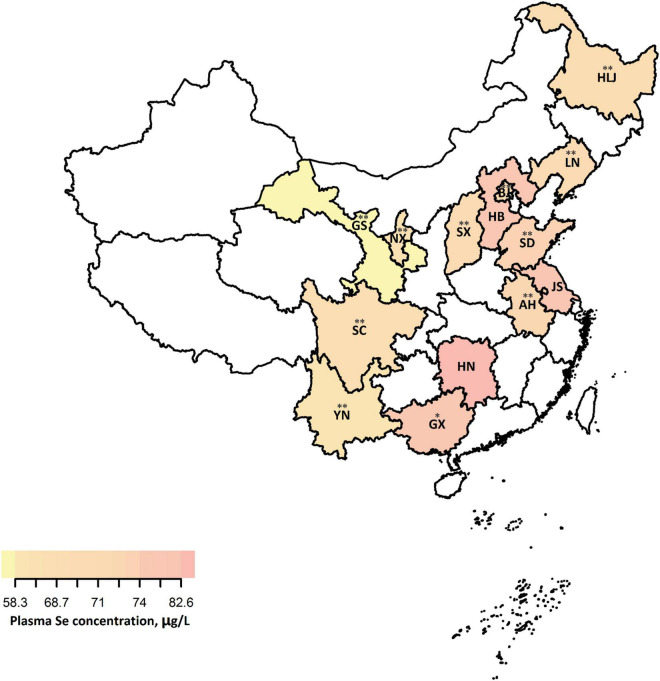
Regional distribution patterns of plasma Se concentrations (μg/L) in Chinese middle-aged and elderly adults with hypertension^1^. ^1^Plasma Se concentrations were adjusted for sex, age, BMI, SBP, DBP, history of hypertension, antihypertensive drug use, multivitamin use, smoking, alcohol drinking, meat consumption, and consumption of fruits and vegetables. *p < 0.05, ^**^p < 0.001, the average plasma Se concentration of this province significantly differed from that of the province with the relatively highest plasma Se status (Hunan, 88.6 μg/L). The 14 provinces in our study including AH, Anhui (*n* = 127); BJ, Beijing (*n* = 197); GC, Gansu (*n* = 185); GX, Guangxi (*n* = 200); HB, Hebei (*n* = 200); HLJ, Heilongjiang (*n* = 179); HN, Hunan (*n* = 200); JS, Jiangsu (*n* = 200); LN, Liaoning (*n* = 200); NX, Ningxia (*n* = 182); SC, Sichuan (*n* = 200); SD, Shandong (*n* = 172); SX, Shanxi (*n* = 196); and YN, Yunnan (*n* = 161).

**FIGURE 2:**
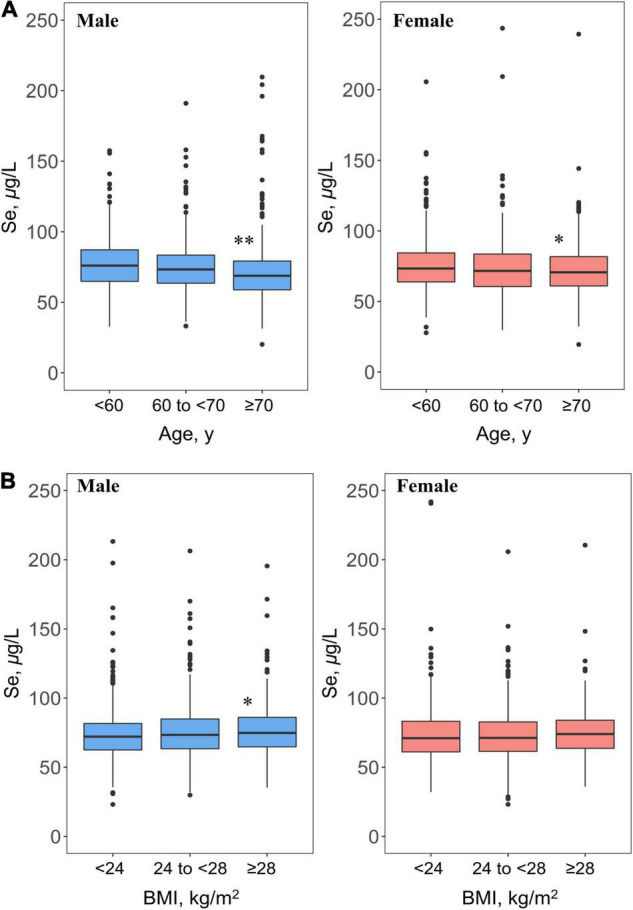
Age **(A)** and BMI **(B)** distribution patterns of plasma Se concentrations (μg/L) in Chinese middle-aged and elderly adults with hypertension^1^. ^1^If not stratified, plasma Se concentrations were adjusted for sex, age, region (Se-marginal, Se-sufficient, and Se-rich areas), BMI, SBP, DBP, history of hypertension, antihypertensive drug use, multivitamin use, smoking, alcohol drinking, meat consumption, and consumption of fruits and vegetables. *p < 0.05, **p < 0.001, the average plasma Se concentration of this group significantly differed from those aged less than 60 years or with BMI less than 24 kg/m^2^ among male and female participants.

Overall, there were significant regional, sex, age, and BMI differences in plasma Se status among the participants with hypertension from 14 provinces of China. The regional distribution pattern of plasma Se status is presented in Figure 1. In provinces of Se-marginal regions, including Gansu, Heilongjiang, Liaoning, and Shandong, participants had relatively lower mean values of plasma Se concentrations, ranging from 58.3 (Gansu) to 76.1 (Shandong) μg/L. For those living in Se-sufficient regions (Anhui, Beijing, Hebei, Jiangsu, Ningxia, Shanxi, Sichuan, and Yunnan), relatively low plasma Se concentrations (mean values ranging from 67.3 for Yunnan to 83.2 for Jiangsu, μg/L) were found. In addition, significantly higher plasma Se concentrations were found in Se-rich regions, including Guangxi (mean value, 81.7 μg/L) and Hunan (mean value, 88.6 μg/L) (Supplementary Table 1). The results comparing plasma Se concentrations among different sexes, age groups, and BMI groups were also explored in this study. After controlling for several potential determinants, including demographic characteristics and lifestyle, men tended to have relatively higher average plasma Se concentrations than females (*p* = 0.082) (Supplementary Table 3). The differences in plasma Se status between men and women by province, age, and BMI is also shown (Figure 2 and Supplementary Table 4). An age pattern in regards to plasma Se distribution, was found with a significant decline in Se concentration by age (men: for trend, *p* < 0.001, for non-linearity, *p* = 0.784; women: for trend, *p* < 0.001, for non-linearity, *p* = 0.160) (Figure 2A and Table 2). Moreover, plasma Se concentrations increased with elevated BMI, especially in men (for trend = 0.023, for non-linearity, *p* = 0.209) (Figure 2B and Table 2).

**TABLE 2:** The β and 95% CI of plasma Se concentrations by demographic factors in Chinese middle-aged and elderly adults with hypertension stratified by sex.

Variables	N	Plasma Se, μ g/L	Crude Model*		Adjusted Model^†^	
			β (95% CI)	*p*	β (95% CI)	*p*
**Male**						
**Age, years**						
<60	597	79.4 ± 19.0	0.00 (Ref)		0.00 (Ref)	
60 to <70	353	76.1 ± 20.3	−3.35 (−6.08, −0.62)	0.016	−2.30 (−5.02, 0.42)	0.097
≥70	439	71.2 ± 23.3	−8.17 (−10.73, −5.61)	<0.001	−6.65 (−9.51, −3.78)	<0.001
*p*, for trend				<0.001		<0.001
**Region**						
Se-rich	227	89.3 ± 18.5	0.00 (Ref)		0.00 (Ref)	
Se-sufficient	779	75.1 ± 18.7	−14.15 (−17.12, −11.18)	<0.001	−15.02 (−18.29, −11.75)	<0.001
Se-marginal	383	69.8 ± 23.4	−19.50 (−22.79, −16.20)	<0.001	−19.82 (−23.44, −16.21)	<0.001
*p*, for trend				<0.001		<0.001
**BMI, kg/m^2^**						
≥28	267	78.2 ± 20.7	0.00 (Ref)		0.00 (Ref)	
24 to <28	624	76.4 ± 20.2	−1.82 (−4.83, 1.19)	0.237	−2.09 (−4.94, 0.76)	0.150
<24	498	74.2 ± 22.1	−3.99 (−7.12, −0.87)	0.012	−3.59 (−6.67, −0.50)	0.022
*p*, for trend				0.009		0.023
**Female**						
**Age, years**						
<60	459	75.0 ± 19.9	0.00 (Ref)		0.00 (Ref)	
60 to <70	311	72.9 ± 22.9	−2.11 (−5.14, 0.91)	0.170	−2.65 (−5.56, 0.25)	0.073
≥70	440	69.7 ± 20.6	−5.33 (−8.07, −2.58)	<0.001	−4.79 (−7.59, −2.00)	<0.001
*p*, for trend				<0.001		<0.001
**Region**						
Se-rich	173	88.4 ± 16.3	0.00 (Ref)		0.00 (Ref)	
Se-sufficient	684	72.7 ± 20.2	−15.71 (−19.01, −12.41)	<0.001	−15.92 (−19.60, −12.24)	<0.001
Se-marginal	353	64.5 ± 20.4	−23.95 (−27.54, −20.35)	<0.001	−21.77 (−25.81, −17.72)	<0.001
*p*, for trend				<0.001		<0.001
**BMI, kg/m^2^**						
≥28	226	74.2 ± 19.7	0.00 (Ref)		0.00 (Ref)	
24 to <28	488	71.8 ± 20.5	−2.40 (−5.72, 0.93)	0.237	−2.96 (−6.06, 0.14)	0.061
<24	496	72.6 ± 22.3	−1.65 (−4.96, 1.67)	0.012	−3.07 (−6.22, 0.08)	0.056
*p*, for trend				0.492		0.093

*For continuous variables, the values are presented as mean ± SD.*

*^1^Se-marginal areas in China include four provinces (Gansu, Heilongjiang, Liaoning, and Shandong), Se-sufficient areas in China include eight provinces (Anhui, Beijing, Hebei, Jiangsu, Ningxia, Shanxi, Sichuan, and Yunnan), and Se-rich areas in China include two provinces (Guangxi and Hunan).*

*Values for β and 95% CI are from multilinear regression analysis.

*^†^If not stratified, the model was adjusted for sex, age, region (Se-marginal, Se-sufficient, and Se-rich areas), BMI, SBP, DBP, history of hypertension, antihypertensive drug use, multivitamin use, smoking, alcohol drinking, meat consumption, and consumption of fruits and vegetables.*

**FIGURE 3:**
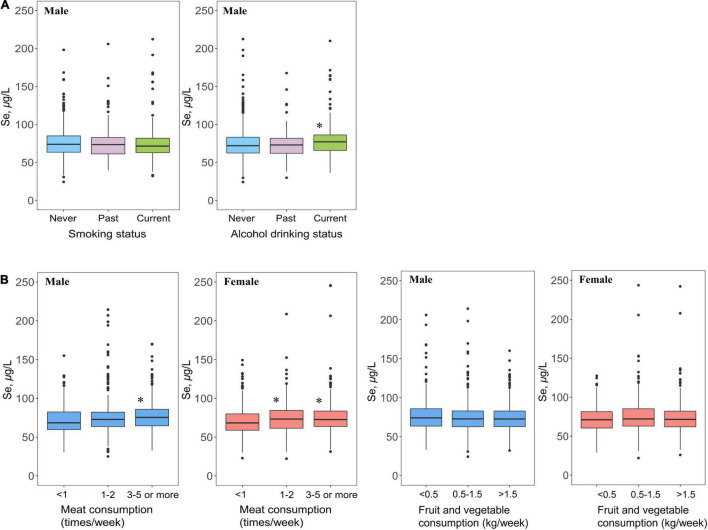
Lifestyle **(A)** and dietary habits **(B)** distribution patterns of plasma Se concentrations (μg/L) in Chinese middle-aged and elderly adults with hypertension^1^. ^1^If not stratified, plasma Se concentrations were adjusted for sex, age, region (Se-marginal, Se-sufficient, and Se-rich areas), BMI, SBP, DBP, history of hypertension, antihypertensive drug use, multivitamin use, smoking, alcohol drinking, meat consumption, and consumption of fruits and vegetables. *p < 0.05, ^**^p < 0.001, the average plasma Se concentration of this group significantly differed from non-smokers, those consuming meat less than 1 time/week or consuming fruits and vegetables less than 0.5 kg/week among male and female participants.

The lifestyle distribution patterns of plasma Se status among men and women are shown in Figure 3. Current male alcohol drinkers had significantly higher average plasma Se concentrations than male non-alcohol drinkers (*p* < 0.05). However, current male smokers tended to have lower average plasma Se concentrations than male non-smokers (*p* = 0.177) (Figure 3A and Supplementary Table 5). Moreover, the average plasma Se concentrations significantly increased with higher frequency in meat in both men and women (for trend, *p* < 0.05) (Figure 3B and Table 3).

**TABLE 3:** The β and 95% CI of plasma Se concentrations by lifestyle and dietary habits in Chinese middle-aged and elderly adults with hypertension stratified by sex^1^.

Variables	*N*	Plasma Se, μ g/L	Crude Model *		Adjusted Model^†^	
			β (95% CI)	*p*	β (95% CI)	*p*
**Male**						
**Smoking status**						
Never	1820	74.0 ± 21.1	0.00 (Ref)		0.00 (Ref)	
Past	271	74.7 ± 22.2	−0.83 (−3.90, 2.23)	0.594	−1.61 (−4.74, 1.52)	0.313
Current	508	75.5 ± 20.7	0.50 (−1.97, 2.98)	0.690	−2.53 (−4.98, −0.08)	0.043
*p*, for trend				0.726		0.041
**Alcohol drinking status**						
Never	1948	73.3 ± 21.3	0.00 (Ref)		0.00 (Ref)	
Past	191	74.8 ± 19.6	−0.21 (−3.62, 3.20)	0.905	−0.39 (−3.85, 3.08)	0.826
Current	460	78.8 ± 20.6	4.69 (2.23, 7.15)	<0.001	4.44 (1.97, 6.91)	<0.001
*p*, for trend				<0.001		<0.001
**Meat consumption frequency**						
<1 time/week	252	70.4 ± 20.8	0.00 (Ref)		0.00 (Ref)	
1–2times/week	447	73.5 ± 23.5	3.08 (−0.12, 6.28)	0.059	3.64 (0.55, 6.73)	0.020
3–5 or more times/week	690	79.6 ± 18.7	9.18 (6.19, 12.17)	<0.001	5.31 (2.29, 8.33)	<0.001
*p*, for trend				<0.001		<0.001
**Fruits and vegetables consumption**						
<0.5 kg/week	320	76.8 ± 23.7	0.00 (Ref)		0.00 (Ref)	
0.5–1.5 kg/week	487	74.2 ± 21.1	−2.63 (−5.60, 0.33)	0.081	−2.29 (−5.12, 0.54)	0.112
>1.5 kg/week	582	77.0 ± 19.3	0.20 (−2.66, 3.07)	0.890	−2.85 (−5.63, −0.07)	0.044
*p*, for trend				0.583		0.056
**Female**						
**Meat consumption frequency**						
<1 time/week	369	67.4 ± 19.7	0.00 (Ref)		0.00 (Ref)	
1–2times/week	442	71.8 ± 20.5	4.36 (1.50, 7.21)	0.002	3.35 (0.60, 6.11)	0.017
≥3 times/week	399	78.2 ± 21.8	10.07 (6.22, 13.92)	<0.001	4.74 (1.74, 7.74)	0.002
*p*, for trend				<0.001		0.001
**Fruits and vegetables consumption**						
<0.5 kg/week	273	69.3 ± 18.8	0.00 (Ref)		0.00 (Ref)	
0.5–1.5 kg/week	476	72.9 ± 22.1	3.60 (0.47, 6.73)	0.024	3.17 (0.16, 6.18)	0.039
>1.5 kg/week	461	74.1 ± 21.1	4.75 (1.60, 7.90)	0.003	1.86 (−1.22, 4.93)	0.237
*p*, for trend				0.004		0.384

*For continuous variables, the values are presented as mean ± SD.*

*^1^Se-marginal areas in China include four provinces (Gansu, Heilongjiang, Liaoning, and Shandong), Se-sufficient areas in China include eight provinces (Anhui, Beijing, Hebei, Jiangsu, Ningxia, Shanxi, Sichuan, and Yunnan), and Se-rich areas in China include two provinces (Guangxi and Hunan).*

*Values β and 95% CI are from multilinear regression analysis.

*^†^If not stratified, model was adjusted for sex, age, region (Se-marginal, Se-sufficient, and Se-rich areas), BMI, SBP, DBP, history of hypertension, antihypertensive drug use, multivitamin use, smoking, alcohol drinking, meat consumption, and consumption of fruits and vegetables.*

### Association of Demographic Characteristics and Lifestyle With Plasma Selenium Status

The multivariate regression analysis showed that, for the male participants, after adjusting for relative factors, the lower plasma Se concentrations were significantly associated with age ≥70 years (*p* < 0.001), being from Se-sufficient regions (*p* < 0.001) and Se-marginal regions (*p* < 0.001), and having BMI < 24 kg/m^2^ (*p* = 0.022) (Table 2). Moreover, for males, relatively lower plasma Se concentrations were associated with current smoking (*p* = 0.043) and consumption of fruits and vegetables >1.5 kg/week (*p* = 0.044), whereas with current alcohol drinking (*p* < 0.001), consuming meat 1–2 times/week (*p* = 0.020) and consuming meat 3–5 or more times/week (*p* < 0.001) were associated with relatively higher plasma Se concentrations (Table 3). For the female participants, the lower plasma Se concentrations were associated with age ≥70 years (*p* < 0.001) and being from Se-sufficient regions (*p* < 0.001) and Se-marginal regions (*p* < 0.001) (Table 2). Additionally, relatively higher plasma Se concentrations were associated with consuming meat 1–2 times/week (*p* = 0.017), consuming meat 3–5 or more times/week (*p* = 0.002) and consuming fruits and vegetables 0.5–1.5 kg/week (*p* = 0.039) (Table 3). Furthermore, plasma Se concentrations were inversely associated with baseline SBP (per SD increment, *p* = 0.008; for non-linearity, *p* = 0.312) and baseline DBP (per SD increment, *p* = 0.002; for non-linearity, *p* = 0.757) in the male participants. For the female participants, plasma Se concentrations were inversely associated with baseline DBP (per SD increment, *p* = 0.010; for non-linearity, *p* = 0.428) (Supplementary Table 6).

## Discussion

To our knowledge, our study presents the first nationwide plasma Se distribution obtained using the ICP–MS method in both middle-aged and elderly adults with hypertension in China. Our research provides two new insights. First, in this population, low circulating Se status was remarkably widespread, and plasma Se status varied significantly according to the region, sex, age, and BMI. Second, the lifestyle characteristics including smoking, alcohol drinking, and consumption of meat, fruits, or vegetables all had a close relationship with plasma Se status and may be potential intervenable factors for improvement of circulating Se status in adults with hypertension.

As Se is a trace element with a narrow normal range, maintaining a dynamic balance of Se distribution is imperative to human antioxidant function (1). Relatively high circulating Se concentrations (>120 μg/L on average) have been found among populations in the United States, Japan, Canada, etc. (28, 29). In contrast, the low circulating Se status and multielement imbalances were found to be associated with Keshan disease or Kashin–Beck disease in the northeast and northwest regions of China (21, 30, 31). However, a study conducted among 10 provinces and municipalities of China assessing hair Se concentrations in Chinese inhabitants across the northeast to southeast China indicated that about 84% of all residents had normal hair Se content. The study (32) also discovered sex and geographical differences in hair Se status. Moreover, recent evidence based on the 2015 China Health and Nutrition Survey (CHNS) showed that close to 60% of men’s daily intake of Se is insufficient [not reaching the recommended amount of the estimated average requirement (EAR)] (33). Other studies have focused on Se status among people with various diseases, such as cancer, CVD, diabetes, etc., revealing the differences in Se distribution compared to healthy people (34–36). Overall, the previous studies have examined Se levels and Se intake of people in specific parts of China (37–39), but these studies are limited by the available materials used to measure Se, such as hair, nails, and urine, which do not reflect Se circulation status in the body (19). While a few studies on blood Se status have been conducted, they have been limited in terms of representativeness and sample size (28). As far as we know, there has been no report on the status of circulating Se levels in a large and representative Chinese population with hypertension that is exposed to diverse environmental Se levels.

We found relatively low Se levels and significant regional differences in Se levels in Chinese adults with hypertension. Hypertensive patients may encounter oxidative stress more frequently than healthy subjects and may need more support from nutrients, such as alpha tocopherol (Vit E), ascorbic acid (Vit C), Se, etc., to lower the concentration of reactive oxygen species (ROS). Thus, the relatively low Se levels among these patients may be related to a higher Se demand and consumption based on their special physical conditions (9, 40). Furthermore, the interaction between the origin of foods consumed, dietary habits, and the environment on human Se status could be crucial. The level of Se in food is mostly determined by the amount of Se in the soil. Se cannot be synthesized in the human body. Consequently, the source for Se in the human body almost entirely originates from food intake (41). Cereals, fish, eggs, and meats are well known as the major dietary sources of Se. However, the Se content of the same food was found to be lower in China, compared with that of America, Japan, Canada, etc. (42). Additionally, much of the Chinese population consume more cereals, fruits, or vegetables than they do seafood, fish, eggs, or meat (the main Se-rich food in China) in daily life (43). Thus, we speculated that it was Se-rich food bioavailability, as indicated by environmental Se exposure, along with diverse dietary habits that led to the varied distribution of human plasma Se regionally. From what we have found, the average plasma Se levels of participants in Se-marginal regions were lower, while those in Se-rich regions were higher. It is worth noting that even in Se-sufficient regions, the Se status among participants was not greatly improved, and the Se insufficiency rate exceeded 50%, indicating that Se levels are not only affected by soil Se. Ecological factors, dietary intake, and differences in body metabolism may also be important influences that jointly determine individual Se levels (44).

In this study, it was found that the plasma Se levels of men were slightly higher than that of women, even when the potential effects of other factors were excluded, which was consistent with some previous studies (45, 46), but not with all of them (47). Sexual dimorphism may partially explain the differences observed among males and females regarding Se status (48). This is of fundamental importance, and sex differences should be considered when analyzing the Se status. Se has indeed been demonstrated to play a direct role in testosterone synthesis and in protecting the testes against toxins (49). It has been consistently shown that Se directly influences testosterone production from early development and it may also indirectly enhance spermatogenesis through its regulation of testosterone production (50). To achieve normal physiological functions, men require more Se than women, especially since the retention rate for Se is prominent in the testes (51). This could be one of the key explanations for the Se disparity between men and women. In addition, even when exposed to similar environmental Se levels, the sex differences in Se levels may also be determined by dietary habits and lifestyles, which deserves more relevant research in the future.

With regard to aging, the decline in Se levels in Chinese adults with hypertension was noticeable. The impact of age on Se status needs to be completely considered from two aspects. Less Se-rich food intake could be the first main reason for the poor Se status among the elderly. A nutritional health survey conducted among the Chinese elderly found that residents living in inland areas consumed saltwater fish less than one time per month (52). A large number of elderly Chinese adults tend to be vegetarian, which is detrimental to adequate Se intake. Moreover, the decrease in absorption and metabolism function could partly explain the poor Se status in elderly participants (53).

Higher BMI may be associated with higher plasma Se levels. The plasma Se concentrations of participants with BMI over 28 kg/m^2^ were greater than that of participants with BMI lower than 24 kg/m^2^. The trend remained after adjustment for relevant variables. Previous research findings on the link between BMI and serum Se have been inconsistent. A recent study based on the U.S. Third National Health and Nutrition Examination Survey (NHANES III) found a negative correlation between BMI and serum Se concentrations (46). However, in a nationally representative sample of 1,045 British adults, and in a cohort of 3,387 Danish men aged 53–74, BMI was not associated with circulating Se concentrations (15, 47). The extraordinary effect revealed by BMI in our findings should not be ignored. The current population of elderly people in China has suffered through difficult periods during the establishment and development of modern China. In the initial stages, their amount of primary food intake was insufficient, rendering a focus on their nutritional status to be irrelevant. However, after the food scarcity problem was completely resolved, more attention has been drawn to human health, especially on nutrition. Therefore, people with higher BMI tend to have higher Se levels, which may be linked to high-quality food intake and social-economic status. In general, the relationship between BMI and Se status needs to be verified by more large-scale studies, and our study provides a certain extent of evidence for the Se distribution of hypertensive adults with different BMI levels in China.

Although the relationship between Se and blood pressure or hypertension is still inconclusive, we found plasma Se to be inversely associated with baseline SBP and DBP. Our findings were consistent with that of some previous case–control studies (13, 23). However, the results based on cross-sectional studies are still controversial. One study that included 722 Finnish male adults also found a significant inverse relation between Se and SBP (54), while some other studies in 2,638 middle-aged Americans, 3,041 Danish elderly adults, and 680 Indian adults found a positive association between Se and SBP (14, 15, 55). Moreover, most other studies did not find a significant relationship between Se and blood pressure (7). However, more studies are needed to verify our results and to further examine the biological mechanisms underlying the associations.

The close relation between circulating Se status with lifestyle and dietary habits including smoking, alcohol drinking, and meat, fruit, and vegetable consumption, was also found among adults with hypertension. Our finding that male smokers tended to have relatively lower plasma Se levels compared to male non-smokers is consistent with previous results. A study conducted among 44 adults in a seleniferous area of America indicated that smokers had lower tissue Se concentrations compared to non-smokers due, at least in part, to lower Se intake (56). However, there is other evidence that smokers may have reduced food intake and tend to select diets of low nutrient density. Male smokers tended to have a higher frequency of meat and fruit and vegetable consumption and lower BMI than non-smokers in the current study, suggesting that the lower Se concentrations of smokers may be determined by multiple factors. Alcohol intake was found to be associated with elevated serum Se levels in 1,183 Japanese and 124 Americans (57, 58). Similarly, we discovered that male alcohol drinkers had considerably higher plasma Se levels. The results from NHANES 2003–2008 showed that men consumed an excess of non-alcohol sourced energy on drinking days relative to non-drinking days. Specifically, men consumed more from the meat, poultry, and fish group, and in particular, more meat (59). Accordingly, male alcohol drinkers in the current study, tended to consume more meat, suggesting that the higher Se concentrations observed in male alcohol drinkers may be partly explained by their relatively higher Se-rich food intake (Supplementary Table 5). The typical food sources with the highest Se concentrations (mg/kg) are seafood and organ meats, followed by muscle meats and crops, dairy products and milk, grain and cereals, fruits, and vegetables (42). As expected, in our study, higher circulating Se levels were found in men or women with the greatest consumption of meat. However, greater consumption of fruits and vegetables seemed to be associated with relatively lower plasma Se levels for men, which may relate to the low Se content in fruits and vegetables and personal dietary structures.

Several limitations of this study merit consideration when interpreting our conclusions. First, our study did not cover all 31 provinces throughout the country. However, the study population was from 14 populated provinces in China, each with diverse Se content in the soil. The results should be interpreted with caution due to the small sample size in some categories when the data were stratified by sex and soil Se distribution. Second, we lacked detailed data on potential confounding factors such as estimated dietary Se information, Se concentrations of food groups, information on Se supplementation, etc. Third, the results of this study were based on a population of middle-aged and elderly Chinese adults with hypertension, thus the results might not be representative of other ethnic groups. Fourth, due to limitations of the initial design, we were not able to test the data of GPX activity and SELENOP, which are important functional biomarkers of Se. Further characterization of these biomarkers will aid in revealing the functional part of Se status in adults with hypertension. Moreover, the type of Se compound is now considered relevant in the assessment of its relation to human health and diseases (60–62). Thus, the determination of different forms of Se, such as inorganic selenium and organic Se, would help to better assess Se status and its association with related diseases in future studies. Owing to these limitations, our findings need to be confirmed in future studies with larger sample sizes and more Se-related biomarkers. In addition, studies are required to further evaluate dietary structures and other lifestyle factors that influence circulating Se status.

## Conclusion

In conclusion, using a validated ICP–MS method, we found significant evidence for low plasma Se concentrations and a high prevalence of Se insufficiency among Chinese adults with hypertension. Women, the elderly (age ≥ 60 years), those with lower BMI (<28 kg/m^2^), and those living in Se-marginal and Se-sufficient regions of China had significantly lower plasma Se levels. Our findings revealed the importance of environmental, demographic, and lifestyle factors on human Se status. Additionally, we found that proper alcohol intake and increased consumption of Se-rich food (such as meat) may contribute to helping maintain relatively higher circulating Se levels among Chinese middle-aged and elderly patients with hypertension to meet the demands for antioxidation.

## Data Availability Statement

The raw data supporting the conclusions of this article will be made available by the authors, without undue reservation.

## Ethics Statement

The studies involving human participants were reviewed and approved by the Ethics Committee of Peking University First Hospital, Beijing, China. The patients/participants provided their written informed consent to participate in this study.

## Author Contributions

ZW, XX, XQ, and HG: conceptualization. XX, XQ, CL, and ZW: methodology. LL and CL: software. XQ, CL, and YW: validation. ZW, TL, and YL: formal analysis. ZW, TL, YW, YS, LL, ZZ, XH, PC, YL, BW, JL, YZ, YH, and HZ: investigation. XX, XQ, YL, XH, and HG: resources. LL, TL, and CL: data curation. ZW, XQ, and HG: manuscript (original draft) preparation. XX and YH: review and editing of manuscripts. XX, XQ, and HG: supervision. ZW and XX: project administration. XX: funding acquisition. All authors have read the final version of the manuscript and have given final approval for this version of the manuscript to be published.

## Conflict of Interest

The authors declare that the research was conducted in the absence of any commercial or financial relationships that could be construed as a potential conflict of interest.

## Publisher’s Note

All claims expressed in this article are solely those of the authors and do not necessarily represent those of their affiliated organizations, or those of the publisher, the editors and the reviewers. Any product that may be evaluated in this article, or claim that may be made by its manufacturer, is not guaranteed or endorsed by the publisher.

## Footnotes

1
http://www.R-project.org/

2
http://www.resdc.cn

3
http://www.empowerstats.com

## Abbreviations

βs: beta coefficients
BMI: body mass index
CHNS: China Health and Nutrition Survey
Cis: confidence intervals
CVD: cardiovascular disease
DBP: diastolic blood pressure
EAR: estimated average requirement
GPX: glutathione peroxidase
ICP–MS: inductively coupled plasma mass spectrometry
NHANES: National Health and Nutrition Examination Survey
PRs: prevalence ratios
ROS: reactive oxygen species
SBP: systolic blood pressure
Se: selenium
SELENOP: selenoprotein P
tHcy: total homocysteine
WHO: World Health Organization
IQR: interquartile range.
