# Supplementary material for: A Cross-Sectional Study of the Distribution Patterns and Potential Determinants in Plasma Selenium Status Among Chinese Adults With Hypertension
Source: Front Nutr. 2022 May 17;9:882309. doi: 10.3389/fnut.2022.882309 (PMC9152156; doi:10.3389/fnut.2022.882309)
Supplement: Supplementary file 1 [file Data_Sheet_1.docx]

**Supplementary Material**

| **Characteristics** | **Total ^2^** | **Provinces** | | | | | | | | | | | | | |
| --- | --- | --- | --- | --- | --- | --- | --- | --- | --- | --- | --- | --- | --- | --- | --- |
|  |  | **Anhui** | **Beijing** | **Gansu** | **Guangxi** | **Hebei** | **Heilongjiang** | **Hunan** | **Jiangsu** | **Liaoning** | **Ningxia** | **Shandong** | **Shanxi** | **Sichuan** | **Yunnan** |
| N | 2599 | 127 | 197 | 185 | 200 | 200 | 179 | 200 | 200 | 200 | 182 | 172 | 196 | 200 | 161 |
| Male, *n* (%) | 1389 (53.4) | 75 (59.1) | 125 (63.5) | 94 (50.8) | 115 (57.5) | 111 (55.5) | 88 (49.2) | 112 (56.0) | 113 (56.5) | 113 (56.5) | 88 (48.4) | 88 (51.2) | 95 (48.5) | 91 (45.5) | 81 (50.3) |
| Age, y |  |  |  |  |  |  |  |  |  |  |  |  |  |  |  |
| < 60 | 1056 (40.6) | 24 (18.9) | 81 (41.1) | 77 (41.6) | 75 (37.5) | 85 (42.5) | 79 (44.1) | 88 (44.0) | 72 (36.0) | 84 (42.0) | 80 (44.0) | 66 (38.4) | 84 (42.9) | 81 (40.5) | 80 (49.7) |
| ≥ 60, < 70 | 664 (25.6) | 40 (31.5) | 51 (25.9) | 51 (27.6) | 60 (30.0) | 53 (26.5) | 40 (22.3) | 53 (26.5) | 49 (24.5) | 48 (24.0) | 40 (22.0) | 39 (22.7) | 51 (26.0) | 49 (24.5) | 40 (24.8) |
| ≥ 70 | 879 (33.8) | 63 (49.6) | 65 (33.0) | 57 (30.8) | 65 (32.5) | 62 (31.0) | 60 (33.5) | 59 (29.5) | 79 (39.5) | 68 (34.0) | 62 (34.1) | 67 (39.0) | 61 (31.1) | 70 (35.0) | 41 (25.5) |
| SBP, mmHg | 141.3 (131.3-153.3) | 136.0 (128.2-144.0) | 135.7 (127.7-145.7) | 154.0 (150.0-157.3) | 142.3 (133.3-152.4) | 140.7 (133.9-152.6) | 142.7 (134.2-155.7) | 143.3 (131.3-155.0) | 137.3 (130.0-145.5) | 142.3 (131.7-156.8) | 140.8 (130.0-151.9) | 139.7 (127.1-151.4) | 137.8 (128.7-150.1) | 146.7 (136.3-158.2) | 138.7 (127.3-149.7) |
| DBP, mmHg | 87.0 (79.7-94.0) | 83.0 (76.5-88.7) | 84.3 (76.3-90.7) | 95.3 (92.0-98.3) | 83.5 (77.3-91.1) | 90.0 (80.7-96.7) | 89.0 (80.8-97.2) | 89.3 (83.6-94.3) | 85.5 (79.2-90.0) | 88.0 (80.7-96.8) | 87.2 (80.0-93.5) | 81.5 (71.9-91.1) | 84.0 (77.6-90.0) | 86.2 (78.3-94.4) | 85.0 (79.0-93.7) |
| Antihypertensive drug use, *n* (%) | 1758 (67.6) | 66 (52.0) | 196 (99.5) | 128 (69.2) | 52 (26.0) | 152 (76.0) | 126 (70.4) | 47 (23.5) | 149 (74.5) | 173 (86.5) | 161 (88.5) | 130 (75.6) | 88 (44.9) | 151 (75.5) | 139 (86.3) |
| Multivitamin use, *n* (%) | 209 (8.0) | 11 (8.7) | 16 (8.1) | 7 (3.8) | 2 (1.0) | 17 (8.5) | 17 (9.5) | 7 (3.5) | 5 (2.5) | 14 (7.0) | 10 (5.5) | 14 (8.1) | 17 (8.7) | 34 (17.0) | 38 (23.6) |
| History of hypertension, *n* (%) | 2367 (91.1) | 127 (100.0) | 192 (97.5) | 64 (34.6) | 181 (90.5) | 187 (93.5) | 163 (91.1) | 171 (85.5) | 191 (95.5) | 196 (98.0) | 178 (97.8) | 169 (98.3) | 193 (98.5) | 199 (99.5) | 156 (96.9) |
| Body mass index, kg/m^2^ |  |  |  |  |  |  |  |  |  |  |  |  |  |  |  |
| < 24 | 994 (38.2) | 55 (43.3) | 56 (28.4) | 70 (37.8) | 102 (51.0) | 57 (28.5) | 57 (31.8) | 100 (50.0) | 72 (36.0) | 66 (33.0) | 58 (31.9) | 49 (28.5) | 83 (42.3) | 105 (52.5) | 64 (39.8) |
| 24 to < 28 | 1112 (42.8) | 50 (39.4) | 81 (41.1) | 78 (42.2) | 72 (36.0) | 90 (45.0) | 88 (49.2) | 75 (37.5) | 98 (49.0) | 81 (40.5) | 94 (51.6) | 70 (40.7) | 85 (43.4) | 72 (36.0) | 78 (48.4) |
| ≥ 28 | 493 (19.0) | 22 (17.3) | 60 (30.5) | 37 (20.0) | 26 (13.0) | 53 (26.5) | 34 (19.0) | 25 (12.5) | 30 (15.0) | 53 (26.5) | 30 (16.5) | 53 (30.8) | 28 (14.3) | 23 (11.5) | 19 (11.8) |
| Selenium, *μ*g/L^1^ | 74.4 ± 19.4 | 74.0 ± 14.2 | 72.2 ± 14.7 | 58.3 ± 20.5 | 81.7 ± 17.6 | 82.6 ± 19.2 | 71.0 ± 20.4 | 88.6 ± 18.8 | 83.2 ± 15.9 | 72.0 ± 19.2 | 68.7 ± 19.1 | 76.1 ± 18.6 | 71.8 ± 14.4 | 70.5 ± 24.2 | 67.3 ± 22.8 |
| Smoking status, *n* (%) |  |  |  |  |  |  |  |  |  |  |  |  |  |  |  |
| Never | 1820 (70.0) | 79 (62.2) | 96 (48.7) | 141 (76.2) | 142 (71.0) | 150 (75.0) | 137 (76.5) | 144 (72.0) | 153 (76.5) | 115 (57.5) | 126 (69.2) | 135 (78.5) | 143 (73.0) | 156 (78.0) | 103 (64.0) |
| Past | 271 (10.4) | 22 (17.3) | 47 (23.9) | 5 (2.7) | 8 (4.0) | 25 (12.5) | 15 (8.4) | 19 (9.5) | 12 (6.0) | 22 (11.0) | 28 (15.4) | 0 (0.0) | 22 (11.2) | 13 (6.5) | 33 (20.5) |
| Current | 508 (19.5) | 26 (20.5) | 54 (27.4) | 39 (21.1) | 50 (25.0) | 25 (12.5) | 27 (15.1) | 37 (18.5) | 35 (17.5) | 63 (31.5) | 28 (15.4) | 37 (21.5) | 31 (15.8) | 31 (15.5) | 25 (15.5) |
| Alcohol drinking status, *n* (%) |  |  |  |  |  |  |  |  |  |  |  |  |  |  |  |
| Never | 1948 (75.0) | 85 (66.9) | 120 (60.9) | 164 (88.6) | 170 (85.0) | 147 (73.5) | 134 (74.9) | 142 (71.0) | 164 (82.0) | 135 (67.5) | 124 (68.1) | 131 (76.2) | 166 (84.7) | 146 (73.0) | 120 (74.5) |
| Past | 191 (7.3) | 18 (14.2) | 24 (12.2) | 7 (3.8) | 5 (2.5) | 16 (8.0) | 12 (6.7) | 28 (14.0) | 3 (1.5) | 13 (6.5) | 24 (13.2) | 0 (0.0) | 9 (4.6) | 14 (7.0) | 18 (11.2) |
| Current | 460 (17.7) | 24 (18.9) | 53 (26.9) | 14 (7.6) | 25 (12.5) | 37 (18.5) | 33 (18.4) | 30 (15.0) | 33 (16.5) | 52 (26.0) | 34 (18.7) | 41 (23.8) | 21 (10.7) | 40 (20.0) | 23 (14.3) |
| Meat consumption frequency, *n* (%) |  |  |  |  |  |  |  |  |  |  |  |  |  |  |  |
| <1 time/week | 621 (23.9) | 15 (11.8) | 48 (24.4) | 34 (18.4) | 6 (3.0) | 73 (36.5) | 41 (22.9) | 26 (13.0) | 35 (17.5) | 73 (36.5) | 29 (15.9) | 61 (35.5) | 76 (38.8) | 77 (38.5) | 27 (16.8) |
| 1-2 times/week | 889 (34.2) | 44 (34.6) | 67 (34.0) | 114 (61.6) | 11 (5.5) | 60 (30.0) | 73 (40.8) | 67 (33.5) | 93 (46.5) | 56 (28.0) | 72 (39.6) | 79 (45.9) | 80 (40.8) | 56 (28.0) | 17 (10.6) |
| 3-5 or more times/week | 1089 (41.9) | 68 (53.5) | 82 (41.6) | 37 (20.0) | 183 (91.5) | 67 (33.5) | 65 (36.3) | 107 (53.5) | 72 (36.0) | 71 (35.5) | 81 (44.5) | 32 (18.6) | 40 (20.4) | 67 (33.5) | 117 (72.7) |
| Fruit and vegetable consumption, *n* (%) |  |  |  |  |  |  |  |  |  |  |  |  |  |  |  |
| <0.5 kg/ week | 593 (22.8) | 19 (15.0) | 21 (10.7) | 23 (12.4) | 25 (12.5) | 80 (40.0) | 28 (15.6) | 59 (29.5) | 19 (9.5) | 34 (17.0) | 24 (13.2) | 98 (57.0) | 34 (17.3) | 57 (28.5) | 72 (44.7) |
| 0.5-1.5 kg/week | 963 (37.1) | 78 (61.4) | 72 (36.5) | 102 (55.1) | 56 (28.0) | 83 (41.5) | 102 (57.0) | 38 (19.0) | 81 (40.5) | 43 (21.5) | 86 (47.3) | 26 (15.1) | 85 (43.4) | 67 (33.5) | 44 (27.3) |
| >1.5 kg/week | 1043 (40.1) | 30 (23.6) | 104 (52.8) | 60 (32.4) | 119 (59.5) | 37 (18.5) | 49 (27.4) | 103 (51.5) | 100 (50.0) | 123 (61.5) | 72 (39.6) | 48 (27.9) | 77 (39.3) | 76 (38.0) | 45 (28.0) |

**Supplemental Table 1. Characteristics of study participants by study provinces *.**

* For continuous variables, values are presented as median (IQR) or mean ± SD.

^1^ Values of plasma Se concentration by provinces were adjusted for sex, age, BMI, SBP, DBP, history of hypertension, antihypertensive drug use, multivitamin use, smoking, alcohol drinking, meat consumption, and consumption of fruits and vegetables.

^2^ Values of plasma Se concentration in total participants were adjusted for sex, age, BMI, region (Se-marginal, Se-sufficient and Se-rich areas), SBP, DBP, history of hypertension, antihypertensive drug use, multivitamin use, smoking, alcohol drinking, meat consumption, and consumption of fruit and vegetable.

**Supplemental Table 2. Baseline characteristics of the selected participants and those not included.**

| **Characteristics** | **First Sampling^1^** | |  | **Second Sampling^2^** | |
| --- | --- | --- | --- | --- | --- |
|  | **Selected** | **Excluded** |  | **Selected** | **Excluded** |
| N | 900 | 5346 |  | 1709 | 18071 |
| Male (%) | 528 (58.7) | 3197 (59.8) |  | 868 (50.8) | 10722 (59.3) |
| Age, y |  |  |  |  |  |
| < 60 | 371 (41.2) | 2168 (40.6) |  | 691 (40.4) | 8412 (46.5) |
| ≥ 60, < 70 | 286 (31.8) | 1745 (32.6) |  | 380 (22.2) | 5896 (32.6) |
| ≥ 70 | 243 (27.0) | 1433 (26.8) |  | 638 (37.3) | 3763 (20.8) |
| SBP, mmHg | 142.3 (131.6-153.7) | 141.7 (132.3-154.0) |  | 140.7 (131.3-153.0) | 142.7 (133.3-154.0) |
| DBP, mmHg | 88.0 (80.0-94.0) | 88.2 (80.0-93.7) |  | 86.7 (79.0-94.0) | 89.0 (80.7-95.3) |
| Antihypertensive  drug use, *n* (%) | 562 (62.4) | 3199 (59.8) |  | 1206 (70.6) | 11733 (64.9) |
| Multivitamin use, *n* (%) | 68 (7.6) | 264 (4.9) |  | 141 (8.3) | 841 (4.7) |
| History of hypertension, *n* (%) | 782 (86.9) | 4639 (86.8) |  | 1593 (93.2) | 17010 (94.1) |
| BMI, kg/m^2^ |  |  |  |  |  |
| < 24 | 357 (39.7) | 2065 (38.6) |  | 641 (37.5) | 6136 (34.0) |
| 24 to < 28 | 383 (42.6) | 2284 (42.7) |  | 731 (42.8) | 8236 (45.6) |
| ≥ 28 | 160 (17.8) | 997 (18.6) |  | 337 (19.7) | 3699 (20.5) |
| Current smoking, *n* (%) | 216 (24.0) | 1175 (22.0) |  | 294 (17.2) | 4090 (22.6) |
| Current alcohol drinking,  *n* (%) | 176 (19.6) | 944 (17.7) |  | 285 (16.7) | 3399 (18.8) |
| Meat consumption frequency, *n* (%) |  |  |  |  |  |
| <1 time/week | 246 (27.3) | 1144 (21.4) |  | 378 (22.1) | 4821 (26.7) |
| 1-2 times/week | 296 (32.9) | 1625 (30.4) |  | 597 (34.9) | 5874 (32.5) |
| 3-5 or more times/week | 358 (39.8) | 2577 (48.2) |  | 734 (42.9) | 7376 (40.8) |
| Fruit and vegetable consumption, *n* (%) |  |  |  |  |  |
| <0.5 kg/ week | 193 (21.4) | 1088 (20.4) |  | 405 (23.7) | 4271 (23.6) |
| 0.5-1.5 kg/week | 291 (32.3) | 1791 (33.5) |  | 673 (39.4) | 6983 (38.6) |
| >1.5 kg/week | 416 (46.2) | 2467 (46.1) |  | 631 (36.9) | 6817 (37.7) |

For continuous variables, values are presented as median (IQR) or mean ± SD.

^1^ First sampling: included 900 participants who were randomly selected from 6246 participants of 9 provinces and stratified by province, and who were enrolled from June to August 2017.

^2^ Second sampling: included 1709 participants who were randomly selected from 19780 participants of 14 provinces (including the 9 provinces in the first sampling set plus an additional 5 provinces) and stratified by province, sex and age groups, and who were enrolled from February 2017 to May 2018.

**Supplemental Table 3. Baseline characteristics of study participants** **stratified by sex.**

| **Characteristics** | **Total ^2^** | **Male** | **Female** | ***P* value** |
| --- | --- | --- | --- | --- |
| N | 2599 | 1389 | 1210 |  |
| Age, y |  |  |  | 0.015 |
| < 60 | 1056 (40.6) | 597 (43.0) | 459 (37.9) |  |
| ≥ 60, < 70 | 664 (25.5) | 353 (25.4) | 311 (25.7) |  |
| ≥ 70 | 879 (33.8) | 439 (31.6) | 440 (36.4) |  |
| Body mass index, kg/m^2^ |  |  |  | 0.021 |
| < 24 | 994 (38.2) | 498 (35.9) | 496 (41.0) |  |
| 24 to < 28 | 1112 (42.8) | 624 (44.9) | 488 (40.3) |  |
| ≥ 28 | 493 (19.0) | 267 (19.2) | 226 (18.7) |  |
| Region ^*^, *n* (%) |  |  |  | 0.307 |
| Se-marginal | 736 (28.3) | 383 (27.6) | 353 (29.2) |  |
| Se-sufficient | 1463 (56.3) | 779 (56.1) | 684 (56.5) |  |
| Se-rich | 400 (15.4) | 227 (16.3) | 173 (14.3) |  |
| Smoking status, *n* (%) |  |  |  | <0.001 |
| Never | 1820 (70.0) | 659 (47.4) | 1161 (96.0) |  |
| Past | 271 (10.4) | 250 (18.0) | 21 (1.7) |  |
| Current | 508 (19.5) | 480 (34.6) | 28 (2.3) |  |
| Alcohol drinking status, *n* (%) |  |  |  | <0.001 |
| Never | 1948 (75.0) | 777 (55.9) | 1171 (96.8) |  |
| Past | 191 (7.3) | 178 (12.8) | 13 (1.1) |  |
| Current | 460 (17.7) | 434 (31.2) | 26 (2.1) |  |
| SBP, mmHg | 141.3 (131.3-153.3) | 140.7 (131.3-151.3) | 142.0 (131.7-154.7) | 0.017 |
| DBP, mmHg | 87.0 (79.7-94.0) | 88.0 (80.0-95.0) | 85.7 (78.3-92.7) | <0.001 |
| Antihypertensive drug use, *n* (%) | 1758 (67.6) | 923 (66.5) | 835 (69.0) | 0.164 |
| Multivitamin use, *n* (%) | 209 (8.0) | 91 (6.6) | 118 (9.8) | 0.003 |
| History of hypertension, *n* (%) | 2367 (91.1) | 1265 (91.1) | 1102 (91.1) | 0.999 |
| Meat consumption frequency, *n* (%) |  |  |  |  |
| <1 time/week | 621 (23.9) | 369 (30.5) | 252 (18.1) | <0.001 |
| 1-2 times/week | 889 (34.2) | 442 (36.5) | 447 (32.2) |  |
| 3-5 or more times/week | 1089 (41.9) | 399 (33.0) | 690 (49.7) |  |
| Fruit and vegetable consumption, *n* (%) |  |  |  | 0.061 |
| <0.5 kg/ week | 593 (22.8) | 320 (23.0) | 273 (22.6) |  |
| 0.5-1.5 kg/week | 963 (37.1) | 487 (35.1) | 476 (39.3) |  |
| >1.5 kg/week | 1043 (40.1) | 582 (41.9) | 461 (38.1) |  |
| Selenium, *μ*g/L^1^ | 74.4 ± 19.4 | 75.0 ± 19.4 | 73.7 ± 19.4 | 0.082 |

For continuous variables, values are presented as median (IQR) or mean ± SD.

Differences in characteristics were compared using t tests for continuous variables and chi-square tests for categorical variables.

^*^ Se-marginal areas in China included 4 provinces (Gansu, Heilongjiang, Liaoning, and Shandong), Se-sufficient areas in China included 8 provinces (Anhui, Beijing, Hebei, Jiangsu, Ningxia, Shanxi, Sichuan, and Yunnan), and Se-rich areas in China included 2 provinces (Guangxi and Hunan).

^1^ Values of plasma Se concentration by sex were adjusted for region (Se-marginal, Se-sufficient and Se-rich areas), age, BMI, SBP, DBP, history of hypertension, antihypertensive drug use, multivitamin use, smoking, alcohol drinking, meat consumption, and consumption of fruit and vegetable.

^2^ Values of plasma Se concentration in total participants were adjusted for sex, age, BMI, region (Se-marginal, Se-sufficient and Se-rich areas), SBP, DBP, history of hypertension, antihypertensive drug use, multivitamin use, smoking, alcohol drinking, meat consumption, and consumption of fruit and vegetable.

**Supplemental Table 4. Distribution of plasma selenium (Se) by province and sex in Chinese middle-aged and elderly adults with hypertension.**

| **Province** | **Male** | | **Female** | |
| --- | --- | --- | --- | --- |
|  | **N** | **Selenium concentration, mean ± SD *μ*g/L** | **N** | **Selenium concentration, mean ± SD *μ*g/L** |
| Anhui | 75 | 73.4 ± 12.7 | 52 | 74.7 ± 16.3 |
| Beijing | 125 | 72.8 ± 16.2 | 72 | 71.3 ± 11.8 |
| Gansu | 94 | 61.8 ± 25.4 ^*^ | 91 | 54.8 ± 12.9 |
| Guangxi | 115 | 81.1 ± 17.3 | 85 | 82.7 ± 17.9 |
| Hebei | 111 | 84.7 ± 21.4 | 89 | 80.2 ± 16.2 |
| Heilongjiang | 88 | 72.5 ± 19.9 | 91 | 69.5 ± 21.0 |
| Hunan | 112 | 88.7 ± 20.2 | 88 | 88.4 ± 17.0 |
| Jiangsu | 113 | 81.2 ± 15.4 ^*^ | 87 | 86.2 ± 15.8 |
| Liaoning | 113 | 72.5 ± 19.5 | 87 | 71.2 ± 19.0 |
| Ningxia | 88 | 70.7 ± 21.8 | 94 | 66.4 ± 16.1 |
| Shandong | 88 | 76.6 ± 20.2 | 84 | 75.8 ± 16.9 |
| Shanxi | 95 | 71.6 ± 13.8 | 101 | 71.9 ± 15.1 |
| Sichuan | 91 | 70.1 ± 17.1 | 109 | 70.9 ± 28.8 |
| Yunnan | 81 | 65.7 ± 20.6 | 80 | 68.7 ± 24.8 |
| Overall ^2^ | 1389 | 75.0 ± 19.4 | 1210 | 73.7 ± 19.4 |

^1^ Values were adjusted for age, BMI, SBP, DBP, history of hypertension, antihypertensive drug use, multivitamin use, smoking, alcohol drinking, meat consumption, and fruit and vegetable consumption.

^2^ Overall values were adjusted for age, region (Se-marginal, Se-sufficient and Se-rich areas), BMI, SBP, DBP, history of hypertension, antihypertensive drug use, multivitamin use, smoking, alcohol drinking, meat consumption, and consumption of fruit and vegetable.

^*^ *P* <0.05, values signiﬁcantly differed among males and females from the same province.

**Supplemental Table 5. Baseline characteristics of male participants stratified by smoking and alcohol drinking status.**

| **Characteristics** | **Smoking status** | | | **Alcohol drinking status** | | |
| --- | --- | --- | --- | --- | --- | --- |
|  | **Never** | **Past** | **Current** | **Never** | **Past** | **Current** |
| N | 659 | 250 | 480 | 777 | 178 | 434 |
| Age, y, *n* (%) |  |  |  |  |  |  |
| < 60 | 254 (38.5) | 83 (33.2) | 260 (54.2) | 280 (36.0) | 69 (38.8) | 248 (57.1) |
| ≥ 60, < 70 | 140 (21.2) | 75 (30.0) | 138 (28.7) | 197 (25.4) | 47 (26.4) | 109 (25.1) |
| ≥ 70 | 265 (40.2) | 92 (36.8) | 82 (17.1) | 300 (38.6) | 62 (34.8) | 77 (17.7) |
| SBP, mmHg | 141.3 (131.7-151.7) | 138.3 (129.3-149.2) | 141.7 (131.6-151.3) | 140.7 (132.3-151.7) | 140.2 (130.0-151.2) | 141.3 (130.0-151.3) |
| DBP, mmHg | 88.3 (80.0-95.0) | 85.5 (79.1-93.5) | 89.3 (81.3-96.4) | 87.7 (80.0-93.7) | 87.7 (80.7-95.3 | 89.3 (80.7-97.6) |
| Antihypertensive drug use, *n* (%) | 417 (63.3) | 198 (79.2) | 308 (64.2) | 506 (65.1) | 134 (75.3) | 283 (65.2) |
| Multivitamin use, *n* (%) | 48 (7.3) | 22 (8.8) | 21 (4.4) | 52 (6.7) | 15 (8.4) | 24 (5.5) |
| History of hypertension, *n* (%) | 584 (88.6) | 245 (98.0) | 436 (90.8) | 678 (87.3) | 170 (95.5) | 417 (96.1) |
| BMI, kg/m^2^, *n* (%) |  |  |  |  |  |  |
| < 24 | 240 (36.4) | 81 (32.4) | 177 (36.9) | 297 (38.2) | 59 (33.1) | 142 (32.7) |
| 24 to < 28 | 292 (44.3) | 116 (46.4) | 216 (45.0) | 348 (44.8) | 79 (44.4) | 197 (45.4) |
| ≥ 28 | 127 (19.3) | 53 (21.2) | 87 (18.1) | 132 (17.0) | 40 (22.5) | 95 (21.9) |
| Region ^*^, *n* (%) |  |  |  |  |  |  |
| Se-marginal | 204 (31.0) | 35 (14.0) | 144 (30.0) | 225 (29.0) | 31 (17.4) | 127 (29.3) |
| Se-sufficient | 340 (51.6) | 189 (75.6) | 250 (52.1) | 404 (52.0) | 121 (68.0) | 254 (58.5) |
| Se-rich | 115 (17.5) | 26 (10.4) | 86 (17.9) | 148 (19.0) | 26 (14.6) | 53 (12.2) |
| Meat consumption frequency, *n* (%) |  |  |  |  |  |  |
| <1 time/week | 132 (20.0) | 44 (17.6) | 76 (15.8) | 158 (20.3) | 22 (12.4) | 72 (16.6) |
| 1-2 times/week | 241 (36.6) | 75 (30.0) | 131 (27.3) | 276 (35.5) | 56 (31.5) | 115 (26.5) |
| 3-5 or more times/week | 286 (43.4) | 131 (52.4) | 273 (56.9) | 343 (44.1) | 100 (56.2) | 247 (56.9) |
| Fruit and vegetable consumption, *n* (%) |  |  |  |  |  |  |
| <0.5 kg/ week | 145 (22.0) | 65 (26.0) | 110 (22.9) | 169 (21.8) | 37 (20.8) | 114 (26.3) |
| 0.5-1.5 kg/week | 245 (37.2) | 92 (36.8) | 150 (31.2) | 295 (38.0) | 59 (33.1) | 133 (30.6) |
| >1.5 kg/week | 269 (40.8) | 93 (37.2) | 220 (45.8) | 313 (40.3) | 82 (46.1) | 187 (43.1) |
| Selenium, *μ*g/L^1^ | 75.8 ± 19.0 | 74.3 ± 21.0 | 73.7 ± 19.1 | 74.3 ± 19.6 | 73.2 ± 18.5 | 78.1 ± 19.4 |

For continuous variables, values are presented as median (IQR) or mean ± SD.

^*^ Se-marginal areas in China include 4 provinces (Gansu, Heilongjiang, Liaoning, and Shandong), Se-sufficient areas in China include 8 provinces (Anhui, Beijing, Hebei, Jiangsu, Ningxia, Shanxi, Sichuan, and Yunnan), and Se-rich areas in China include 2 provinces (Guangxi and Hunan).

^1^ If not stratified, values of plasma Se concentration were adjusted for sex, region (Se-marginal, Se-sufficient and Se-rich areas), age, BMI, SBP, DBP, history of hypertension, antihypertensive drug use, multivitamin use, smoking, alcohol drinking, meat consumption, and consumption of fruit and vegetable.

**Supplemental Table 6. Association between plasma selenium and blood pressure in Chinese middle-aged and elderly adults with hypertension.**

| **Se, *μ*g/L** | **N** | **Crude model** | | **Adjusted model ^1^** | |
| --- | --- | --- | --- | --- | --- |
|  |  | **β (95% CI)** | ***P* value** | **β (95% CI)** | ***P* value** |
| **Male** |  |  |  |  |  |
| SBP, mmHg |  |  |  |  |  |
| per SD increment | 1389 | -1.62 (-2.47, -0.76) | <0.001 | -1.24 (-2.16, -0.33) | 0.008 |
| Quartiles |  |  |  |  |  |
| Q1 (<60.8) | 312 | 0.00 (Ref) |  | 0.00 (Ref) |  |
| Q2 (60.8 to <72.9) | 337 | -2.96 (-5.45, -0.46) | 0.020 | -1.51 (-4.09, 1.07) | 0.251 |
| Q3 (72.9 to <85.2) | 363 | -4.07 (-6.52, -1.63) | 0.001 | -2.56 (-5.14, 0.02) | 0.052 |
| Q4 (≥85.2) | 377 | -5.32 (-7.75, -2.89) | <0.001 | -3.99 (-6.66, -1.31) | 0.004 |
| *P* for trend |  |  | <0.001 |  | 0.003 |
| DBP, mmHg |  |  |  |  |  |
| per SD increment | 1389 | -0.34 (-0.95, 0.27) | 0.273 | -0.95 (-1.54, -0.35) | 0.002 |
| Quartiles |  |  |  |  |  |
| Q1 (<60.8) | 312 | 0.00 (Ref) |  | 0.00 (Ref) |  |
| Q2 (60.8 to <72.9) | 337 | -1.04 (-2.81, 0.73) | 0.250 | -1.38 (-3.06, 0.29) | 0.106 |
| Q3 (72.9 to <85.2) | 363 | -0.49 (-2.22, 1.25) | 0.583 | -1.82 (-3.49, -0.14) | 0.034 |
| Q4 (≥85.2) | 377 | -0.54 (-2.26, 1.19) | 0.541 | -2.51 (-4.24, -0.78) | 0.005 |
| *P* for trend |  |  | 0.737 |  | 0.005 |
| **Female** |  |  |  |  |  |
| SBP, mmHg |  |  |  |  |  |
| per SD increment | 1210 | -1.19 (-2.18, -0.20) | 0.018 | -0.90 (-1.95, 0.16) | 0.096 |
| Quartiles |  |  |  |  |  |
| Q1 (<60.8) | 338 | 0.00 (Ref) |  | 0.00 (Ref) |  |
| Q2 (60.8 to <72.9) | 312 | -3.06 (-5.75, -0.37) | 0.026 | -1.50 (-4.23, 1.22) | 0.280 |
| Q3 (72.9 to <85.2) | 287 | -3.01 (-5.76, -0.25) | 0.033 | -1.65 (-4.48, 1.18) | 0.253 |
| Q4 (≥85.2) | 273 | -2.78 (-5.57, 0.01) | 0.051 | -2.08 (-5.13, 0.96) | 0.180 |
| *P* for trend |  |  | 0.052 |  | 0.185 |
| DBP, mmHg |  |  |  |  |  |
| per SD increment | 1210 | -0.55 (-1.19, 0.09) | 0.093 | -0.85 (-1.50, -0.20) | 0.010 |
| Quartiles |  |  |  |  |  |
| Q1 (<60.8) | 338 | 0.00 (Ref) |  | 0.00 (Ref) |  |
| Q2 (60.8 to <72.9) | 312 | -1.80 (-3.54, -0.05) | 0.044 | -1.81 (-3.49, -0.13) | 0.035 |
| Q3 (72.9 to <85.2) | 287 | -2.74 (-4.53, -0.95) | 0.003 | -2.85 (-4.59, -1.10) | 0.001 |
| Q4 (≥85.2) | 273 | -0.87 (-2.69, 0.94) | 0.345 | -1.88 (-3.75, -0.00) | 0.050 |
| *P* for trend |  |  | 0.166 |  | 0.020 |

^1^ Model was adjusted for age, region (Se-marginal, Se-sufficient and Se-rich areas), BMI, history of hypertension, antihypertensive drug use, multivitamin use, smoking, alcohol drinking, meat consumption, and consumption of fruit and vegetable.





**Supplemental Figure. 1. Flow chart of the study participants.**

^*^ Sample 1 includes 9 provinces in China (Beijing, Gansu, Guangxi, Hebei, Hunan, Jiangsu, Liaoning, Shanxi, and Sichuan)

^†^ Sample 2 includes 14 provinces in China (Anhui, Beijing, Gansu, Guangxi, Hebei, Heilongjiang, Hunan, Liaoning, Jiangsu, Ningxia, Shandong, Shanxi, Sichuan, and Yunnan)

^‡^ Individuals in Sampling population 1 were totally included in Sampling population 2, except for those 900 individuals who had been sampled for Sample 1.


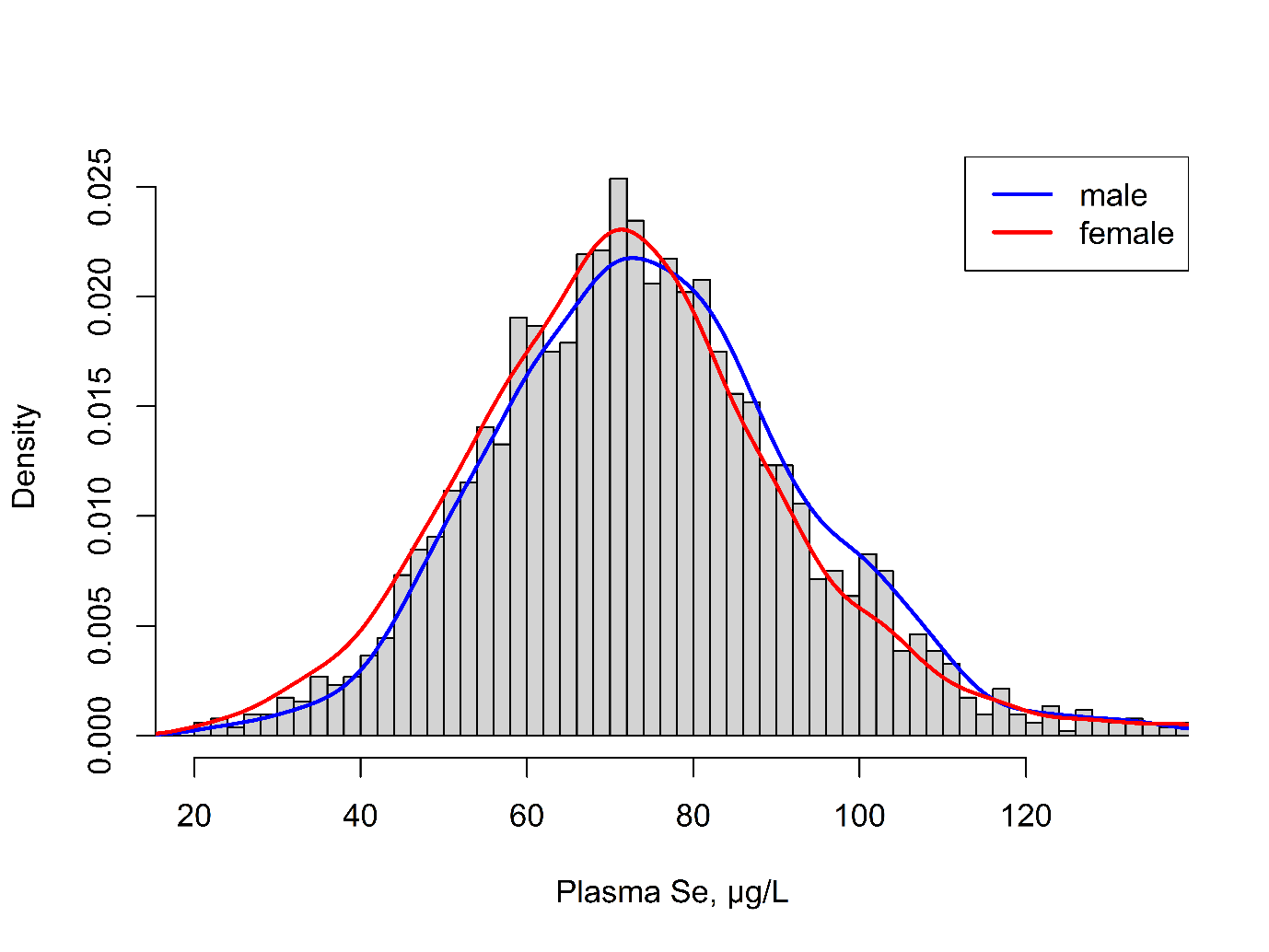


**Supplemental Figure. 2. Plasma selenium distributions (*μ*g/L) in Chinese adults with hypertension (male, *n*=1389; female, *n*=1210).**
